# Numerical Study of Stud Welding Temperature Fields on Steel–Concrete Composite Bridges

**DOI:** 10.3390/ma18153491

**Published:** 2025-07-25

**Authors:** Sicong Wei, Han Su, Xu Han, Heyuan Zhou, Sen Liu

**Affiliations:** 1 Research Institute of Highway, Ministry of Transportation, Beijing 100088, China; fcbwsc@outlook.com (S.W.); han.xu@rioh.cn (X.H.); 2 School of Civil Engineering, Beijing Jiaotong University, Beijing 100091, China; hansu@bjtu.edu.cn (H.S.); 23111312@bjtu.edu.cn (S.L.)

**Keywords:** composite bridges, studs, welding, temperature fields, numerical study

## Abstract

Non-uniform temperature fields are developed during the welding of studs in steel–concrete composite bridges. Due to uneven thermal expansion and reversible solid-state phase transformations between ferrite/martensite and austenite structures within the materials, residual stresses are induced, which ultimately degrades the mechanical performance of the structure. For a better understanding of the influence on steel–concrete composite bridges’ structural behavior by residual stress, accurate simulation of the spatio-temporal temperature distribution during stud welding under practical engineering conditions is critical. This study introduces a precise simulation method for temperature evolution during stud welding, in which the Gaussian heat source model was applied. The simulated results were validated by real welding temperature fields measured by the infrared thermography technique. The maximum error between the measured and simulated peak temperatures was 5%, demonstrating good agreement between the measured and simulated temperature distributions. Sensitivity analyses on input current and plate thickness were conducted. The results showed a positive correlation between peak temperature and input current. With lower input current, flatter temperature gradients were observed in both the transverse and thickness directions of the steel plate. Additionally, plate thickness exhibited minimal influence on radial peak temperature, with a maximum observed difference of 130 °C. However, its effect on peak temperature in the thickness direction was significant, yielding a maximum difference of approximately 1000 °C. The thermal influence of group studs was also investigated in this study. The results demonstrated that welding a new stud adjacent to existing ones introduced only minor disturbances to the established temperature field. The maximum peak temperature difference before and after welding was approximately 100 °C.

## 1. Introduction

Steel–concrete composite bridges are increasingly employed in modern highway construction due to their high strength, lightweight design, and rapid assembly, and the concrete component’s superior stiffness and durability. Studs serve as critical components in these structures, enabling effective collaboration between steel and concrete by transferring shear and tensile forces. This ensures coordinated deformation under load, fully leveraging the advantages of composite systems.

Studs are predominantly welded to steel components. However, the localized heating and uneven cooling during welding generate non-uniform temperature fields in and around the weld zone [1,2,3]. These thermal variations, combined with material-specific differences in thermal expansion and phase transformation-induced volumetric changes, lead to internal constraints that produce residual stresses. Such stresses compromise the mechanical performance of the structure. For instance, in orthotropic steel bridge decks, residual stresses could compromise material strength capacity, accelerate structural damage evolution, diminish bridge resilience [4,5,6], reduce fatigue resistance [7,8], accelerate crack initiation and propagation [9,10,11], and ultimately shorten structural fatigue life [12,13].

Numerical simulations of residual stresses rely on thermo-elastoplastic methods [14,15,16,17] and thermo-mechanical sequential coupling approaches [18,19]. Thermo-elastoplastic finite element analysis (FEA) for welding stress fields employs two methods: direct and indirect coupling. The direct coupling method offers high accuracy but requires significant computational resources and time. In contrast, the indirect coupling method substantially improves computational efficiency while maintaining acceptable accuracy. Residual stress FEA models typically begin with a thermal analysis of the welding process to determine the internal temperature field. This temperature field is then used as the initial condition for subsequent mechanical analysis to calculate residual stresses. Consequently, accurate temperature field simulation is foundational to reliable residual stress predictions.

Numerous studies have investigated the influence of welding parameters on temperature fields. Hussein [20] recorded temperature profiles during small-diameter stud welding using infrared thermography and validated weld performance via torsion tests, providing experimental insights for process optimization. Sun et al. [21] examined the effects of heat source geometry and arc efficiency on temperature fields, residual stresses, and deformations, finding that heat-affected zone boundaries depend predominantly on arc efficiency and marginally on heat source width. Li et al. [22] analyzed temperature distributions and molten pool dimensions under varying laser parameters (e.g., focal length, speed, power), revealing inverse correlations between temperature/pool dimensions and focal length/speed, and a positive correlation with power. Nguyen et al. [23] explored process parameters’ effects on molten pool microstructure and formation mechanisms, while Masoud et al. [24] and Tlili et al. [25] studied laser parameter impacts on temperature fields and weld characteristics. These studies consistently demonstrate parameter-dependent thermal behavior. Li et al. [26] further quantified input current parameters’ influence on weld temperature fields, showing that auxiliary pulsed input current significantly enhances mechanical properties by modifying the heat-affected zone.

Despite significant progress in the field, the influence of critical parameters such as plate thickness and grouped stud configurations on the temperature field remains inadequately explored. To address this research gap, this study establishes a novel integrated framework combining finite element modeling of stud welding temperature fields with experimental validation through infrared thermography. Through a systematic parametric study of input current and plate thickness, we provide quantitative insights into how these factors govern temperature field distribution. These findings yield a fundamental understanding of residual stress mechanisms and distributions, thereby elucidating their impact on mechanical performance and fatigue behavior.

## 2. Finite Element Simulation

### 2.1. Theoretical Framework for Welding Temperature Fields

#### 2.1.1. Fundamental Laws of Heat Transfer

Heat transfer during welding follows three primary mechanisms: thermal conduction, convection, and radiation [27]. During welding, heat is transferred to the steel primarily through radiation and convection. Within the steel substrate, heat propagates predominantly via conduction. Simultaneously, heat dissipates from the welded surface through radiation and convection.

1. Thermal Conduction

Thermal conduction refers to the energy transfer from high-temperature to low-temperature regions due to temperature gradients. This process is governed by Fourier’s law of thermal conduction [27]:

(1) q=−kdT/dx

where *q* is the heat flux (W/m^2^), *k* is the thermal conductivity (W/m·K), and *dT*/*dx* is the temperature gradient.

2. Convective Heat Transfer

Convection occurs at the weld surface due to temperature differences between the steel and surrounding gas or liquid. This is modeled using Newton’s law of cooling [27]:

(2) $$q=h(T_{s}-T_{\infty })$$

where *h* is the convective heat transfer coefficient (W/m^2^·K), *T_s_* is the surface temperature, and *T*_∞_ is the ambient temperature.

3. Radiative Heat Transfer

High-temperature surfaces during welding emit thermal radiation, described by the Stefan–Boltzmann law [27]:

(3) $$q=\epsilon \sigma (T_{s}^{4}-T_{\infty }^{4})$$

where *ϵ* is the emissivity, *σ* is the Stefan–Boltzmann constant (5.67 × 10^−8^ W/m^2^·K^4^), and *T* denotes absolute temperature.

#### 2.1.2. Fundamental Equations of Welding Temperature Fields

1. Governing Differential Equation for Heat Conduction

The transient temperature distribution during welding is described by the three-dimensional heat conduction equation [27]:

(4) $$\rho c\frac{\partial T}{\partial t}=\frac{\partial }{\partial x}\left(\lambda \frac{\partial T}{\partial x}\right)+\frac{\partial }{\partial y}\left(\lambda \frac{\partial T}{\partial y}\right)+\frac{\partial }{\partial z}\left(\lambda \frac{\partial T}{\partial z}\right)+Q$$

where

*ρ*: material density (kg/m^3^);

*c*: specific heat capacity (J/kg·K);

*λ*: thermal conductivity (W/m·K);

*Q*: internal heat generation rate (W/m^3^).

2. Thermal Boundary Conditions

Dirichlet Boundary Condition (First Type).

Specifies known surface temperatures [27]:

(5) $$T=T_{w}$$

where T_w_ is the prescribed temperature.

Neumann Boundary Condition (Second Type).

Defines heat flux across the boundary [27]:

(6) $$-\lambda \frac{\partial T}{\partial n}=q$$

where *q* is the imposed heat flux (W/m^2^), and *n* denotes the normal direction.

Robin Boundary Condition (Third Type).

Accounts for convective and radiative heat exchange with the environment [27]:

(7) $$-\lambda \frac{\partial T}{\partial n}=h(T_{s}-T_{\infty }\;)+\epsilon \sigma (T_{s}^{4}-T_{\infty }^{4})$$

where *h* is the convective coefficient, *ϵ* is emissivity, and *T*_∞_ is ambient temperature.

### 2.2. Heat Source Model

Heat exchange during stud welding involves both localized heat input at the stud–steel interface and heat losses throughout the process. The heat source distribution model is currently the primary method for implementing heat input during welding simulations in ABAQUS. These models fall into the following two categories.

Concentrated heat sources: simplified representations such as point, line, or surface heat sources;

Distributed heat sources: spatially resolved models like the Gaussian and double ellipsoid heat source formulations.

1. Point Heat Source Model [28]
  The point heat source model assumes welding arc energy is concentrated at a single point on the workpiece. This model is typically applied to semi-infinite geometric configurations, where heat propagates in three-dimensional space (x, y, z). It is suitable for simulating surface deposition processes on thick plates.
2. Line Heat Source Model [28]
  The line heat source model distributes energy uniformly along the plate thickness direction, perpendicular to the plate plane. Paired with an infinite plate geometry, this model assumes two-dimensional heat propagation and is ideal for simulating full-penetration welding in thin plates.
3. Surface Heat Source Model [28]
  The surface heat source model, used with infinite rod geometries, assumes uniform heat distribution across the rod’s cross-section and unidirectional propagation. This one-dimensional approach is applicable to processes like electrode tip heating and friction welding.

While concentrated heat source models provide accurate temperature predictions away from the heat source, they exhibit significant errors near the source. Notably, the point heat source model creates a mathematical singularity at the center, predicting infinite temperatures—a physical impossibility in real welding scenarios.

4. Gaussian Heat Source Model [29]
  The Gaussian heat source model distributes energy within a circular area following a Gaussian function. It accounts for heat flux in the x and y directions, assuming symmetry and neglecting thickness-direction effects. While valid for shallow molten pools, this simplification introduces errors in deeper weld pools.
5. Double Ellipsoid Heat Source Model [30,31]
  Planar heat source models (e.g., Gaussian) are accurate for shallow welds and low-arc-penetration processes but fail for high-energy welding (e.g., laser, electron beam) due to neglected depth effects. To address this, Goldak et al. proposed the double ellipsoid model, which divides the heat source into front and rear quarter-ellipsoids. Heat flux density follows a Gaussian distribution, peaking at the center and decaying exponentially toward the edges. This formulation better approximates molten pool geometry and energy penetration in thickness-critical applications.

The suitability of different heat source models is summarized in Table 1.

**Figure 1:**
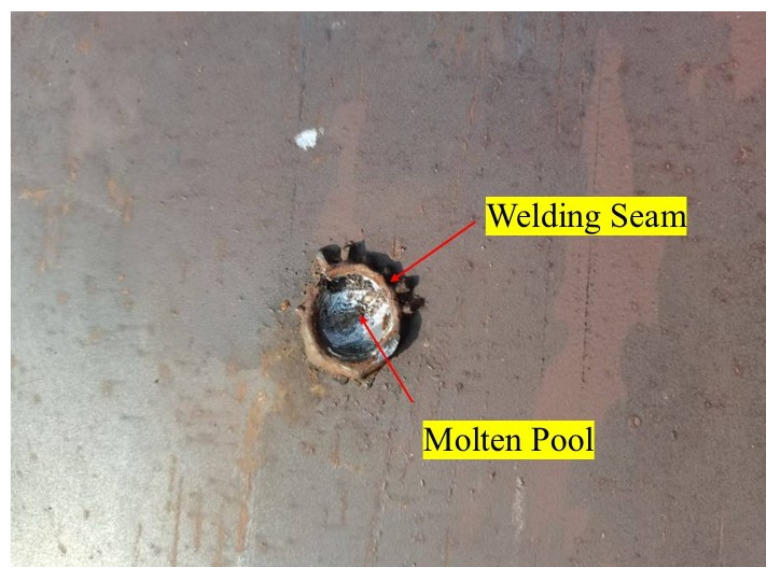
Stud welding test.

**Figure 2:**
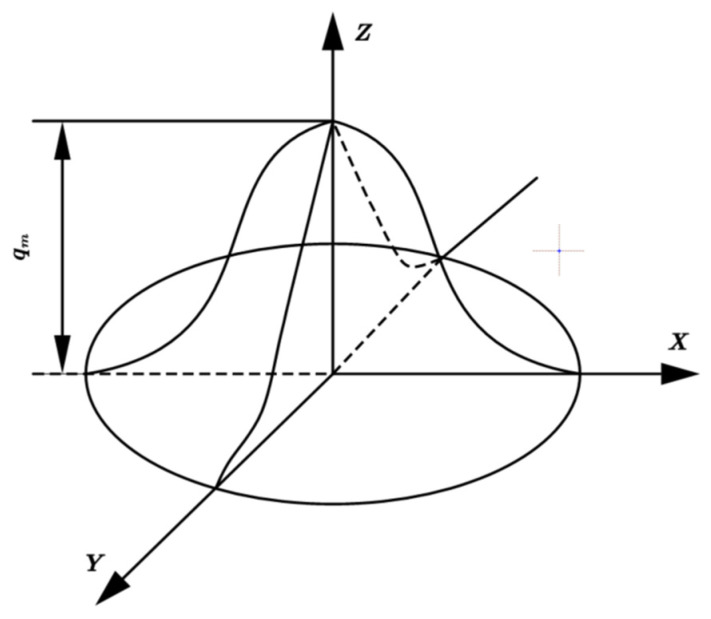
Gaussian heat source model.

Based on experimental results from stud welding tests (see Figure 1), the weld geometry exhibits symmetry with limited molten pool depth. Consequently, variations in heat distribution along the depth direction are tiny, and the Gaussian heat source model is adopted to simulate thermal input current. The schematic of the Gaussian heat source model is illustrated in Figure 2.

It is calculated as follows:

$$q\left(r\right)=q_{m}exp\left(-\frac{3r^{2}}{R^{2}}\right)$$

$$q\left(x,y,t\right)=q_{m}exp\left[-\frac{3\left(x^{2}+y^{2}\right)}{R^{2}}\right]$$

where $q_{m}=\frac{3Q}{\pi R^{2}},Q=\eta UI$.

The Gaussian heat source is modeled as a circular surface heat source. The surface heat flux density *q*(*r*) (W/mm^2^) at radius *r* is illustrated in Figure 2. Here, *q*_m_ denotes the maximum surface heat flux density at the center of the arc heating zone, *R* represents the radius of the heating zone, *Q* is the thermal energy transferred to the workpiece per unit time, *R* denotes the radius of the Gaussian heat source model, r represents the distance from the center of the heat source, *U* and *I* are the arc voltage and input current, respectively, and *η* signifies the arc thermal efficiency.

### 2.3. Finite Element Model Setup

**Figure 3:**
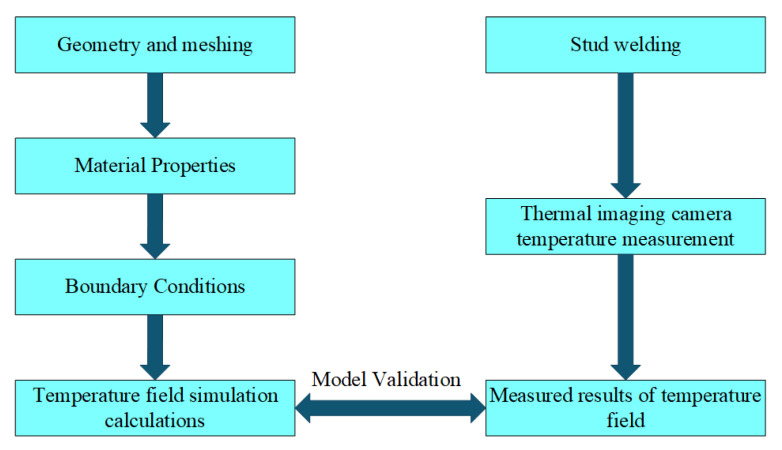
Technical roadmap flow chart.

The detailed flow chart outlining the process from finite element model (FEM) setup to validation is shown in Figure 3.

#### 2.3.1. Geometry and Meshing

**Figure 4:**
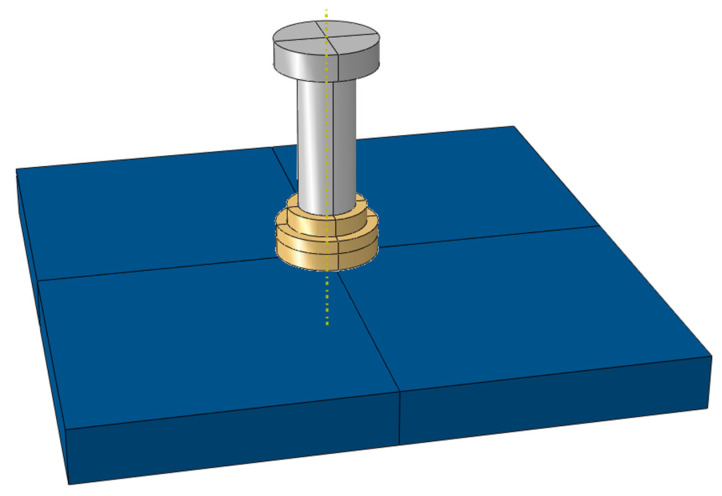
Geometry.

The stud has a diameter of 13 mm, a height of 45 mm, and a cap measuring 23 mm in diameter and 5 mm in height. The weld zone is 18 mm in diameter with a 5 mm height. The steel plate dimensions are 130 mm × 130 mm × 12 mm. The geometric model developed in ABAQUS is shown in Figure 4.

**Figure 5:**
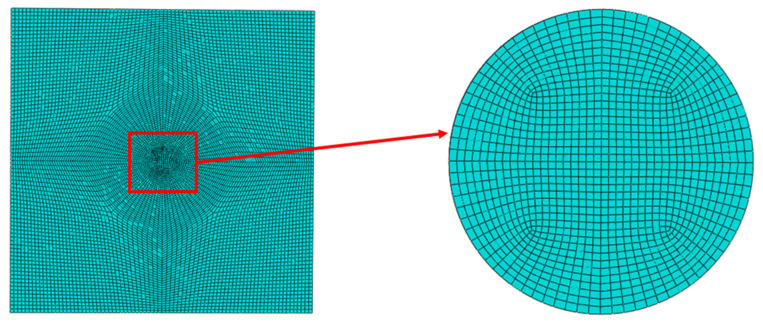
Model discretization approach.

The finite element model employs 8-node linear axisymmetric hexahedral elements (DC3D8) for heat transfer analysis. Mesh convergence analysis (Table 2) indicates that finer meshes in the heat-affected zone yield higher peak transient temperatures. At a 2 mm mesh size, the peak temperature reaches 2014 °C. Further refinement results in small temperature increases, demonstrating diminishing returns on computational accuracy. To balance accuracy and efficiency, a non-uniform mesh strategy is adopted to resolve steep temperature gradients during welding. In detail, a refined mesh size of 2 mm is applied to the weld zone, where temperature and stress gradients are most pronounced. In regions away from the weld, the mesh size gradually transitions to 5 mm as gradients diminish. The final model comprises 151,200 elements, with detailed mesh configuration shown in Figure 5.

#### 2.3.2. Material Properties

**Figure 6:**
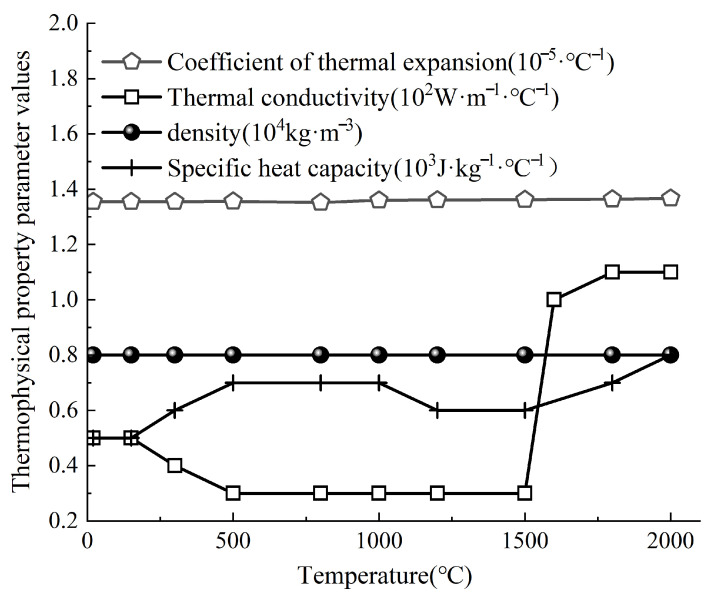
Thermal properties of stud and steel.

The steel material employed in this study was Q345B high-strength low-alloy structural steel. Stud welding involves rapid, high-energy input current, resulting in temperature-dependent variations in the thermal properties of studs and steel. This process constitutes a nonlinear transient analysis. Key material parameters include thermal conductivity, thermal expansion coefficient, specific heat capacity, Poisson’s ratio, and density. Due to limited data availability at extreme temperatures, certain parameters were determined through linear interpolation based on the literature [32,33]. Detailed material properties are provided in Figure 6.

Thermophysical parameters for studs and steel are summarized in Table 3, while those for the ceramic ring [34] are listed in Table 4.

#### 2.3.3. Boundary Conditions

The initial temperature of the structure and environment was set to 25 °C, with absolute zero defined as −273.15 °C. Convective and radiative boundary conditions were applied: convective heat transfer coefficient = 0.15 mW/(mm^2^·K) and emissivity = 0.85 [35,36]. Heat loss primarily occurred at the weld surface. Welding parameters included voltage = 120 V, input current = 1600 A, and welding duration = 2 s. The heat conduction equation is solved by transient heat transfer analysis in ABAQUS to determine temperature fields. The user subroutine DFLUX dynamically defined heat flux density by iterating over all element integration points at each time increment. Key implementation steps include the following.

1. Coordinate transfer: ABAQUS passed integration point coordinates via the COORDS array;
2. Time parameter: Input current step time was transferred through TIME(2);
3. Custom parameters: Heat source power, radius, etc., were incorporated in subroutine or imported via INP files;
4. Radial distance calculation: Distance *r* from each integration point to the heat source center;
5. Heat flux update: Gaussian formula computed *q*(*r*), assigned to FLUX(1) to update localized heat flux.

The analysis used a 2 s heating step and 600 s cooling step.

### 2.4. Model Validation

**Figure 7:**
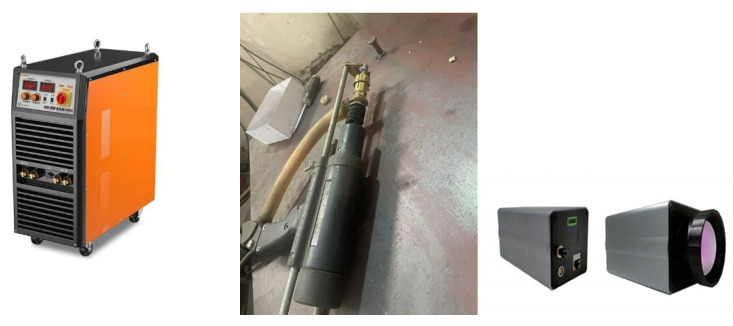
Instruments of stud welding.

The main instruments of stud welding include an arc stud welder (model: RSN2500, China Shandong Tai’an Baokun Electrical Equipment Co., Ltd., Tai’an, China), welding gun, and HiNet-640 thermal imaging camera made by Beijing Hongpu Optoelectronic Technology Co., Ltd., Beijing, China (see Figure 7).

**Figure 8:**
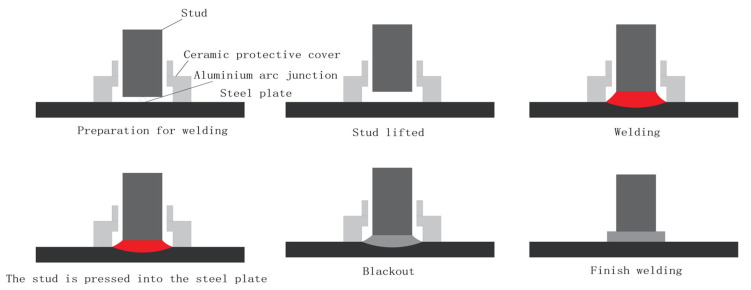
Stud welding process. (The red part represents the thermal energy generated during the welding process, which melts the base material to form a molten pool; finally, it cools down to form the weld seam).

The stud welding process, as shown in Figure 8, includes the following steps.

1. Preparation: Verify equipment functionality; set parameters (input current, voltage, time) based on stud diameter and base material thickness. Clean welding surfaces to remove contaminants.
2. Stud lifting: The gun lifts the stud electromagnetically/pneumatically (2–5 mm height). High voltage ionizes air, generating a 6000–8000 °C arc that melts stud and base metal.
3. Fusion: Molten metal forms a pool, shielded by inert gas (e.g., CO_2_/Ar) to prevent oxidation.
4. Plunge: Post-arc extinction, the stud is forced into the molten pool under mechanical pressure (0.1–0.5 s), expelling slag and gases.
5. Completion: Power is cut; the weld cools naturally to avoid cracking.

**Figure 9:**
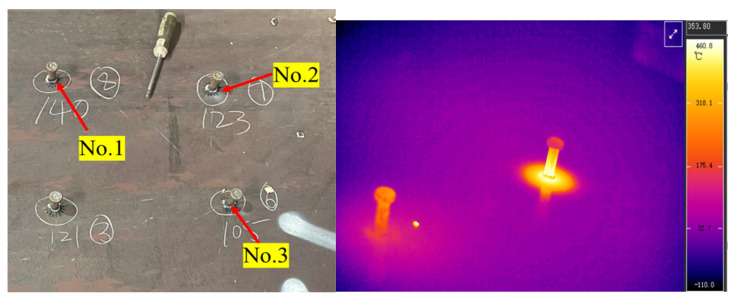
Completed weld.

The final welded studs are shown in Figure 9.

To validate the simulated welding temperature fields, infrared thermography was employed to measure temperatures during the welding process. The infrared camera used in this study has a peak measurement capacity of 2000 °C, an accuracy of ±0.2 °C, and a data acquisition rate of 30 Hz.

**Figure 10:**
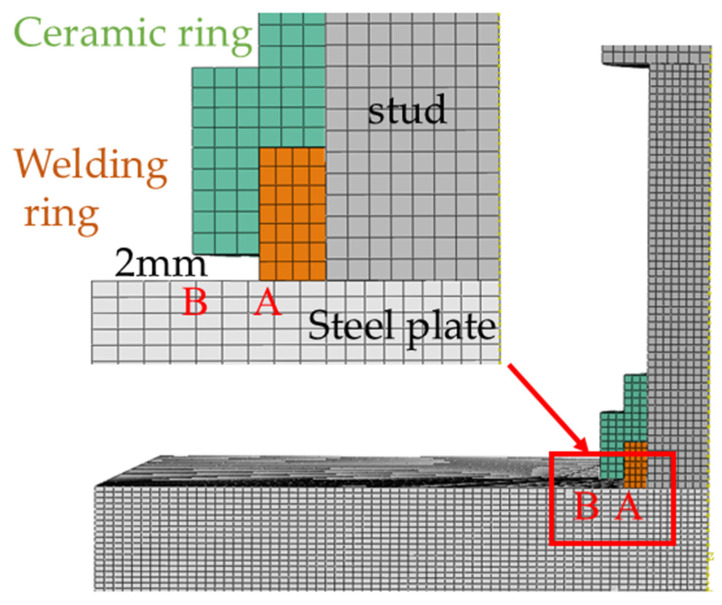
Schematic diagrams of the welding ring (A) and ceramic ring (B).

**Figure 11:**
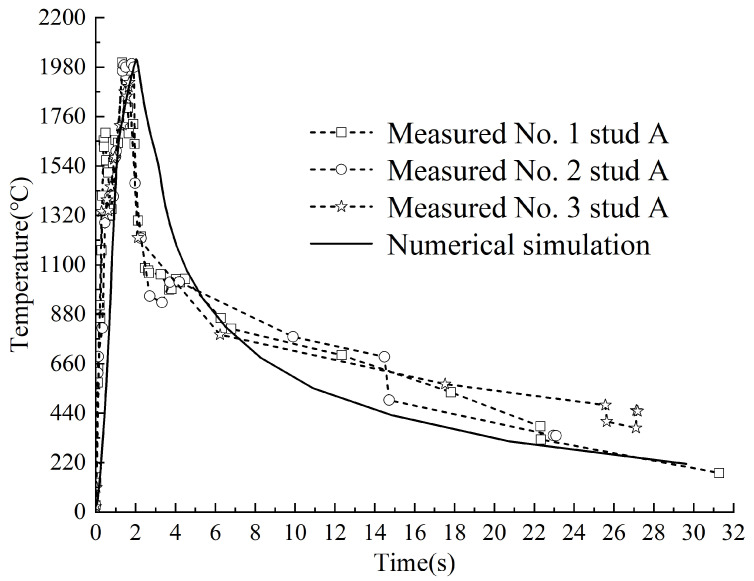
The measured and simulated temperature curves at Stud point A.

**Figure 12:**
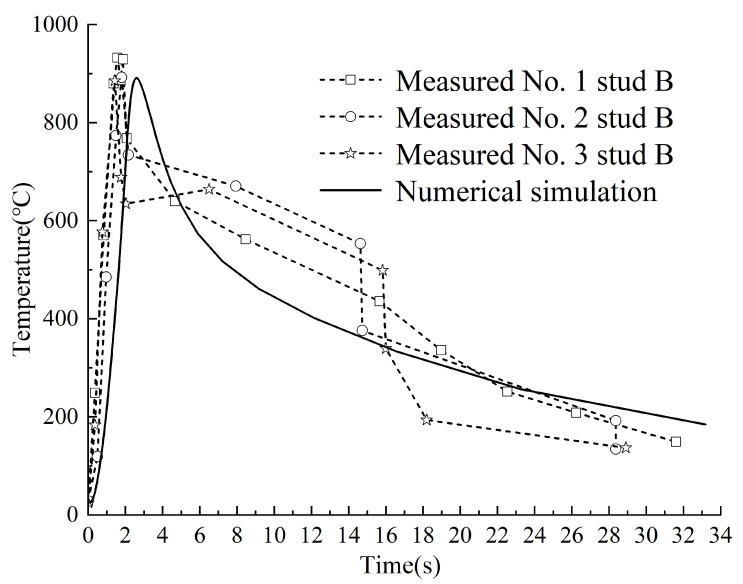
The measured and simulated temperature curves at Stud point B.

A comparative analysis was performed between experimental and simulated temperature profiles at two critical locations: the weld ring (designated as Point A) and the ceramic ring (designated as Point B). The schematic positions of these measurement points are illustrated in Figure 10, while the comparative temperature curves are presented in Figure 11 and Figure 12.

The welding heating phase occurs between 0~2 s, during which the temperature at Stud A reaches its maximum value. Experimental measurements from three Stud A locations yielded peak temperatures of 2000 °C, 1994 °C, and 1911 °C, while the simulated peak temperature at Stud A was 2014 °C, with a maximum error of 5%. The subsequent cooling phase (2~32 s) exhibited a rapid temperature decline between 2~4 s, followed by a gradual reduction in cooling rate as temperatures decreased.

Similarly, Stud B reached its maximum temperature at 2 s during the 0~2 s heating phase. Experimental measurements at three Stud B locations recorded peak temperatures of 930 °C, 892 °C, and 885 °C, closely aligning with the simulated peak temperature of 891 °C, demonstrating tiny error. During the cooling phase (2~32 s), the temperature initially dropped rapidly (2~4 s), then slowed progressively. The experimental final temperature at Stud B stabilized near 140 °C, whereas the simulated result was 184 °C.

Figure 11 and Figure 12 reveal a transient temperature spike in both Stud A and B during cooling, along with minor discrepancies between experimental and simulated temperature curves in this phase.

**Figure 13:**
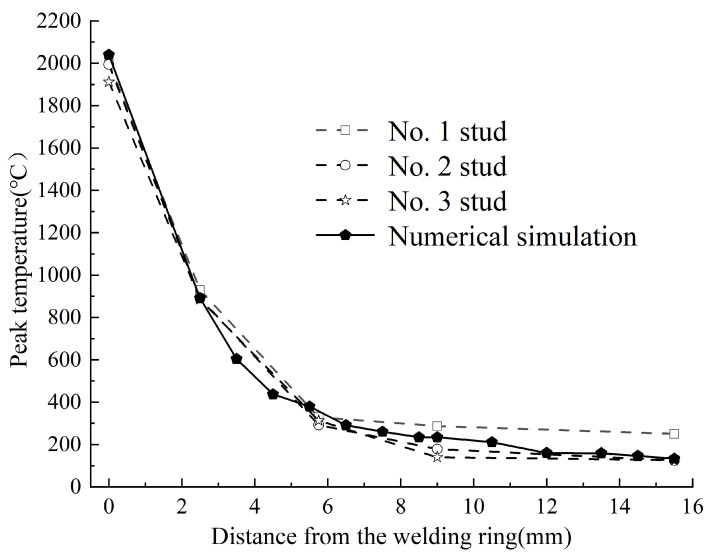
The distribution of the measured transverse temperature field and the simulated temperature field.

For further validation, the experimental and simulated transverse welding temperature fields were compared, as illustrated in Figure 13.

The results indicate close alignment between the measured and simulated temperature fields, with minimal discrepancies. These variations can be attributed to the following two factors.

1. The high-temperature material properties used in the simulation were extrapolated from lower-temperature data, introducing minor inaccuracies;
2. Interference from welding sparks and spatter, as well as the inherent limitations of the temperature measurement equipment.

Despite these factors, the experimental and computational results remain within acceptable margins of error, validating the reliability of the finite element model.

## 3. Stud Welding Temperature Field Results and Analysis

### 3.1. Welding Temperature Field Results

**Figure 14:**
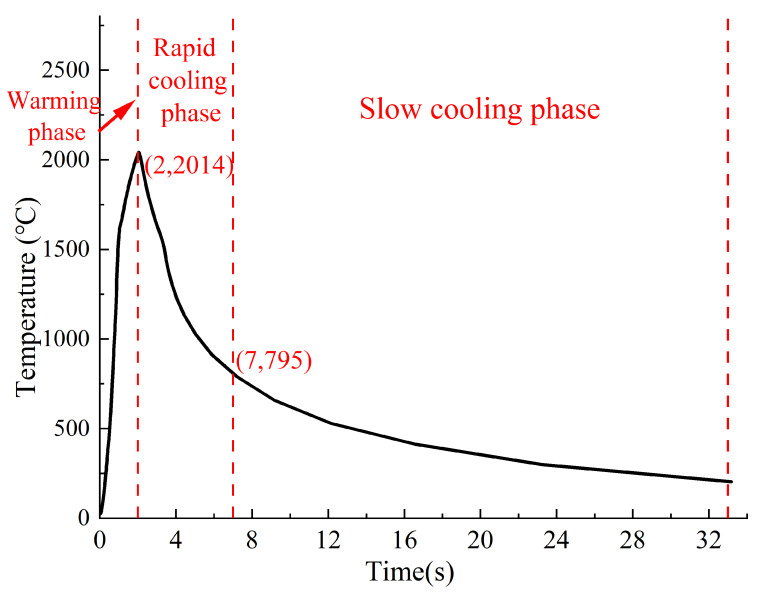
The temperature curve of the welding ring.

Figure 14 presents the temperature curve of the welding ring, divided into three distinct phases: a heating phase (0~2 s), a rapid cooling phase (2~7 s), and a slow cooling phase (7~33 s). Six time points (0, 1, 2, 7, 18, and 33 s) were selected to analyze the temperature evolution.

During the heating phase (0~2 s), the concentrated heat input caused a rapid temperature rise at the weld toe. When the temperature exceeded the material’s solidus temperature (1500 °C), the stud and surrounding steel began to melt, reaching a peak temperature of 2014 °C at the center by the end of this phase (t = 2 s). In the subsequent rapid cooling phase (2~7 s), heat input ceased, and the temperature dropped sharply due to convective and radiative heat transfer between the high-temperature stud/steel and the cooler ambient air. At 7 s, the ceramic ring was removed to facilitate cooling. During the slow cooling phase (7~33 s), the temperature decline gradually decelerated as the system approached thermal equilibrium, stabilizing at 242 °C by 33 s.

**Figure 15:**
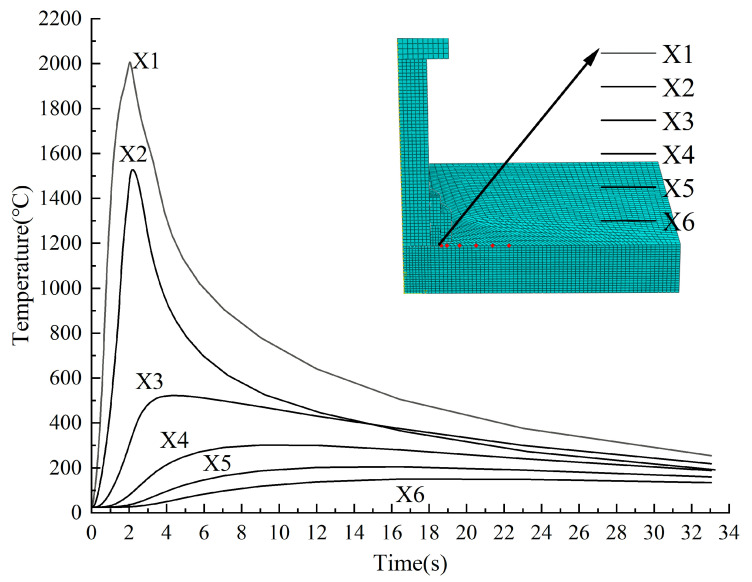
The variations of temperature at transverse positions on the steel surface.

Non-uniform heating and cooling during the welding process result in a complex temperature field [1,2,3] in the weld zone and adjacent areas. The present study focuses on characterizing its spatial distribution and temporal evolution. Figure 15 illustrates the temperature variations at six radial positions (X1~X6) on the steel surface, located 0, 1, 4, 8, 12, and 16 mm from the welding ring edge. Points X1 and X2, closest to the weld, exhibited sharp temperature spikes (2005 °C and 1527 °C, respectively) during the heating phase, followed by a gradual decline. In contrast, the more distant points (X3~X6) showed slower temperature increases without distinct peaks and significantly reduced cooling rates. By the end of the cooling phase, all points converged to temperatures between 135 °C and 260 °C.

Proximity to the welding ring strongly influenced thermal behavior. Near-field points (X1, X2) experienced rapid heating and cooling due to direct heat input, reaching peak temperatures immediately at t = 2 s. For far-field points (X3~X6), peak temperatures decreased with distance, and the time to reach these peaks was delayed due to the time-dependent nature of heat conduction.

**Figure 16:**
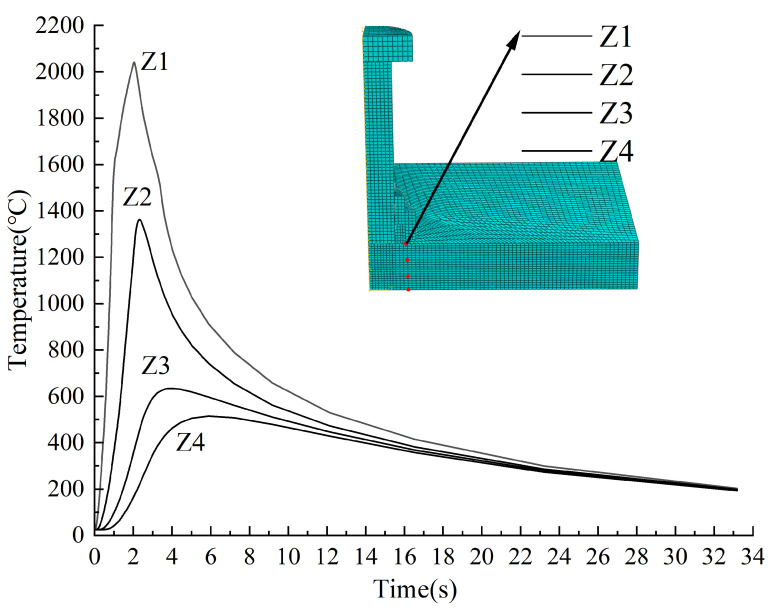
Temperature variation at different positions in the direction of steel thickness.

Figure 16 depicts the temperature changes at four depth-based positions (Z1~Z4) within the steel thickness, located 0, 4, 8, and 12 mm from the welding ring. During the heating phase (0~2 s), points Z1 and Z2 exhibited rapid temperature increase, peaking at 2041 °C and 1362 °C, respectively, with distinct thermal spikes. Cooling ensued thereafter, characterized by a gradual decline in temperature. In contrast, points Z3 and Z4 showed slower heating rates without pronounced peaks, and their cooling rates prior to 10 s were notably lower than those of Z1 and Z2. By the end of the cooling phase, all four points stabilized near 200 °C.

Proximity to the welding ring significantly influenced thermal behavior. Near-field points (Z1, Z2) reached peak temperatures at the end of the heating phase (t = 2 s), followed by steady cooling. For deeper points (Z3, Z4), peak temperatures decreased with distance and were delayed due to the time-dependent nature of heat conduction. These points also displayed a gradual temperature rise and fall, with cooling rates converging to match those of Z1 and Z2 over time.

### 3.2. Effect of Input Current on the Temperature Field

Arc stud welding machines typically operate within an input current range of 40~2500 A [37], as specified by standard product parameters. Input current is a critical process variable, directly influencing the spatiotemporal evolution of welding temperature fields through adjustments in heat input and distribution. However, practical applications rarely exceed 2500 A, and input currents below 900 A fail to achieve the steel’s melting point (1500 °C), as evidenced by the temperature curves. To optimize welding parameters, this study focuses on the 900~2000 A range.

**Figure 17:**
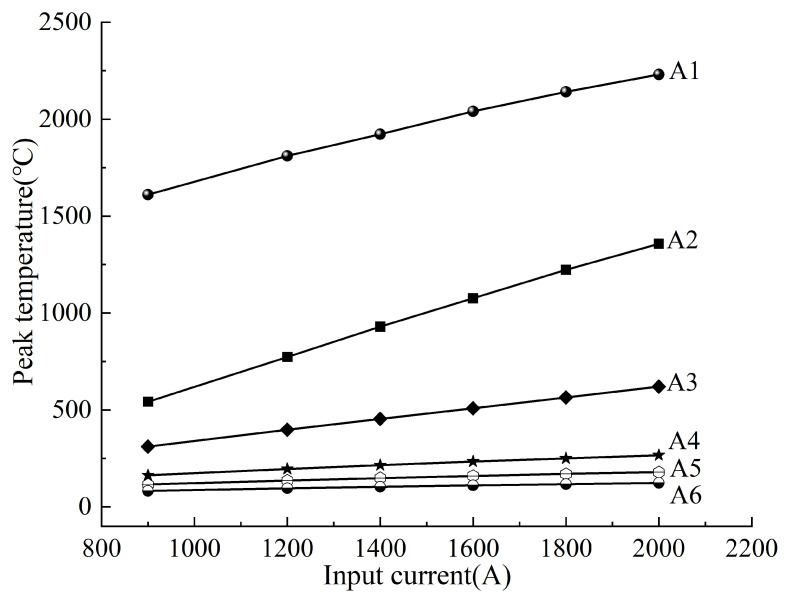
Variation of peak temperature along the transverse direction of the steel under different heating input currents.

Figure 17 illustrates the peak temperatures at six transverse positions (A1~A6) on the steel surface, located 0~17 mm from the welding ring. Increasing the input current from 900 A to 2000 A raised peak temperatures from 1610 °C to 2231 °C at A1 (nearest to the weld) and from 83 °C to 123 °C at A6 (17 mm away). These results confirm a positive correlation between input current and peak temperature, with the effect diminishing significantly beyond 8 mm (A4).

**Figure 18:**
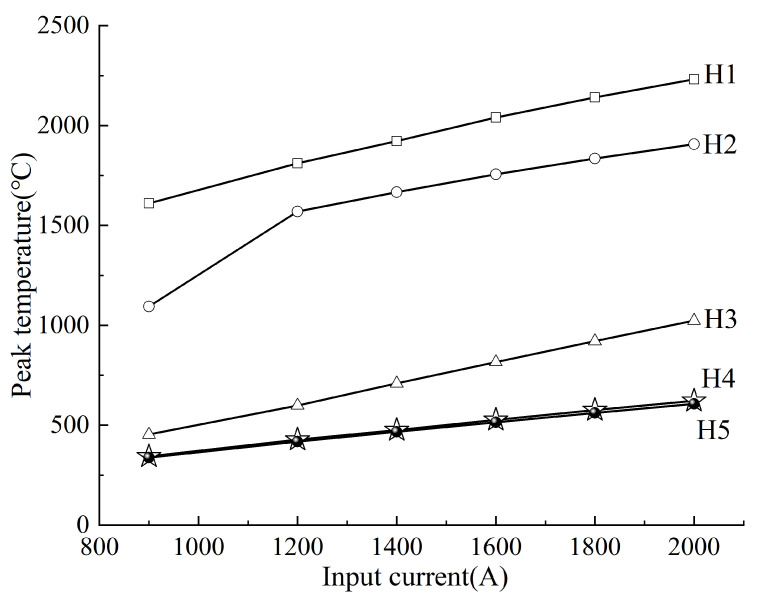
Variation of peak temperature along the thickness of the steel under different heating input currents.

Similarly, Figure 18 shows peak temperatures at five depth-based positions (H1~H5) within the steel thickness. At H1 (surface), temperatures increased from 1610 °C to 2231 °C, while at H5 (12 mm depth), they rose from 337 °C to 607 °C. This trend aligns with the transverse observations, further validating the input current–temperature relationship.

Higher input current increases power input, elevating energy density on the steel surface and enhancing heat absorption [22,23,24]. Consequently, peak temperatures rise proportionally with input current. Notably, lower input current reduces temperature gradients along both transverse and thickness directions, minimizing thermal stress. Thus, using the lowest feasible input current while ensuring weld quality is recommended to balance efficiency and structural stability.

### 3.3. Effect of Plate Thickness on Temperature Field

Steel plate thickness significantly influences the spatio-temporal distribution of temperature fields through its coupling effects on three-dimensional heat conduction pathways, heat dissipation efficiency, and thermal capacity. Thicker plates restrict heat diffusion along the thickness direction, leading to heat accumulation and slower cooling rates. Conversely, thinner plates exhibit steeper temperature gradients due to rapid heat dissipation, which may result in lack of fusion defects or out-of-plane deformation. To investigate these effects, a three-dimensional finite element model was developed to analyze the evolution of peak temperatures in steel plates with thicknesses of 8–28 mm, providing a theoretical basis for optimizing welding processes.

**Figure 19:**
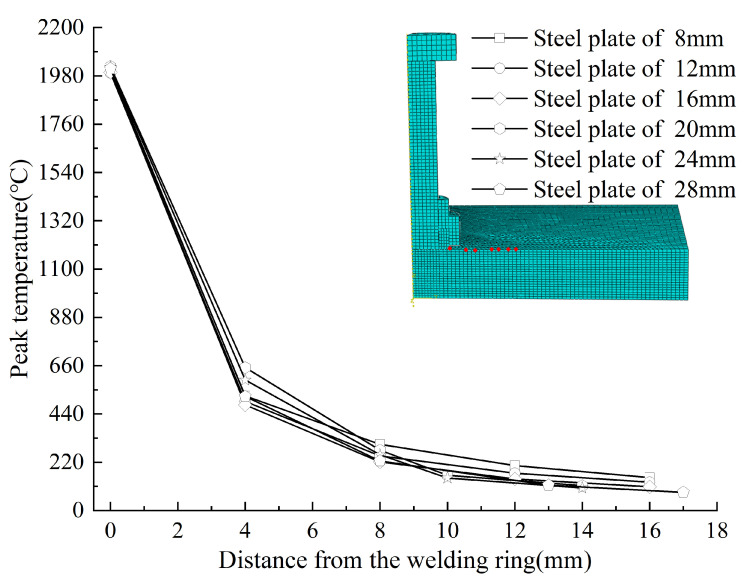
The radial variations of peak temperature across different plate thicknesses.

Figure 19 illustrates the radial variations in peak temperature across different plate thicknesses. Within 0–17 mm of the welding ring, moderate variations (130 °C and 90 °C) were observed at the 4 mm and 8 mm positions, respectively. Overall, however, radial peak temperatures showed minimal sensitivity to plate thickness, indicating its limited-impact on radial thermal behavior.

**Figure 20:**
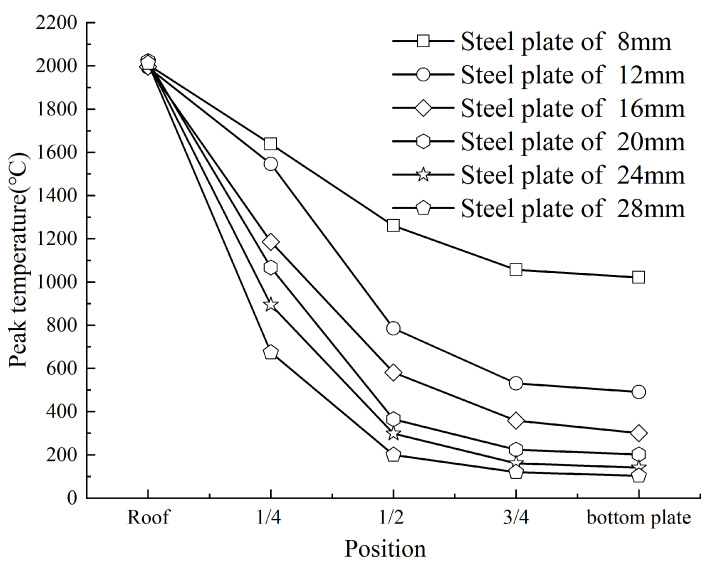
Variation of peak temperature along the steel thickness under different plate thicknesses.

For thickness-direction analysis, plates of 8, 12, 16, 20, 24, and 28 mm were evaluated at five positions: the top surface, 1/4 thickness, mid-thickness, 3/4 thickness, and bottom surface (Figure 20). The top surface consistently maintained a peak temperature of approximately 2000 °C across all thicknesses. In contrast, temperatures at subsurface positions (1/4, 1/2, 3/4 thickness, and bottom surface) decreased with increasing plate thickness. Thinner plates exhibited smaller temperature gradients along the thickness direction, highlighting their more uniform thermal profiles.

### 3.4. Effect of Group Studs on the Temperature Field

In practical construction, multiple studs are often welded consecutively within the same area. During subsequent welding operations, heat generated from these processes may propagate via thermal conduction, exerting secondary thermal effects on the heat-affected zones (HAZs) of previously welded studs and the surrounding steel temperature fields. This dynamic thermal coupling can lead to microstructural degradation in welded joints, altered residual stress distributions, and potential defect formation. However, research on the temperature field superposition effect during multi-stud welding remains limited, particularly regarding how adjacent welding operations influence the thermal history of existing studs. To address this gap, this study employs numerical simulations to investigate the spatio-temporal evolution of temperature fields during multi-stud welding, providing theoretical insights for optimizing welding parameters and ensuring the structural reliability of composite connections.

This work focuses on a two-stud configuration to evaluate the thermal interaction between sequentially welded studs, establishing a foundation for understanding temperature field superposition in multi-stud applications.

In accordance with BS EN 1994-1-1:2004 [38] (Section 3.4.2), the minimum center-to-center spacing between studs in solid slabs must exceed 5d (d = stud diameter) along the shear direction and 2.5d perpendicular to it. For this study, spacings of 100 mm (5d) along the shear direction and 52 mm (4d) perpendicular to it were adopted, aligning with practical engineering standards.

**Figure 21:**
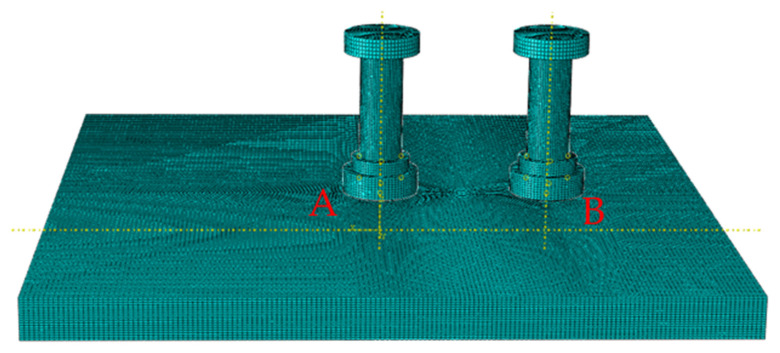
Geometric model.

The stud has a diameter of 13 mm, a height of 45 mm, and a cap measuring 23 mm in diameter and 5 mm in height. The weld zone is 18 mm in diameter with a 5 mm height. The steel plate dimensions are 200 mm × 200 mm × 12 mm, as illustrated in the geometric model (Figure 21).

The numerical simulation adopted an 8-node linear axisymmetric hexahedral element (DC3D8) for heat transfer analysis. The structural material was defined as Q345B high-strength low-alloy steel. Initial thermal boundary conditions were configured as follows: structural initial temperature: 25 °C (with absolute zero set at −273.15°C); convective boundary: heat transfer coefficient = 0.15 mW/(mm^2^·K); and radiative boundary: surface emissivity = 0.85. The heat source model adopts a Gaussian distribution profile, with welding voltage = 120 V and input current = 1600 A. The simulation retained consistent material parameters, boundary conditions, thermal load models, and welding process settings as described in earlier sections. The analysis employed a heating phase time step of 2 s and a cooling phase time step of 600 s. The procedure involved three sequential stages: (1) applying the heat source to Stud A, (2) applying the heat source to Stud B, and (3) allowing natural cooling.

**Figure 22:**
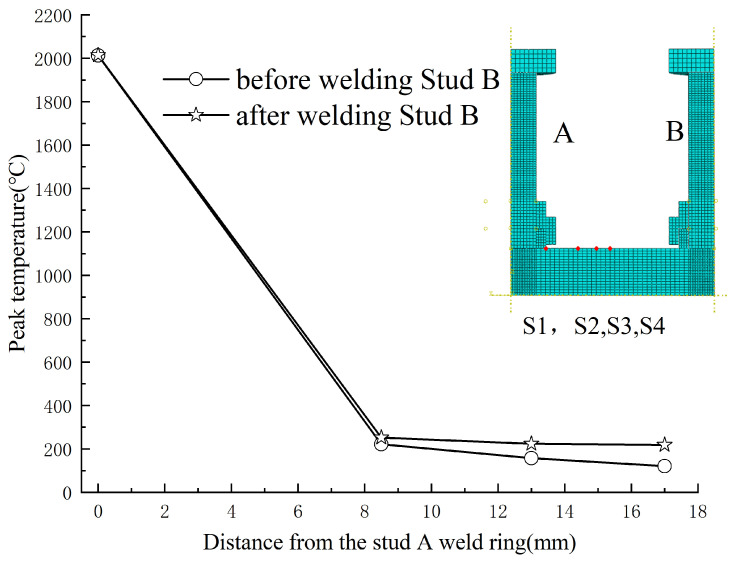
Peak temperature variations at four locations near Stud A before and after welding Stud B.

Four measurement points (S1~S4) were selected at 0, 8.5, 13, and 17 mm from Stud A’s welding ring to evaluate temperature variations before and after welding Stud B. Figure 22 illustrates the peak temperature changes at these points.

Within 0–8 mm of Stud A’s welding ring (S1~S2), the thermal impact of welding Stud B was little, consistent with the localized temperature influence range identified in prior analyses. Beyond 8 mm (S3~S4), the effect intensified with distance, reaching a maximum temperature difference of approximately 100 °C at S4 (17 mm). These findings suggest that subsequent welding operations exert minimal influence on the temperature fields of pre-existing studs, particularly within close proximity.

## 4. Conclusions

A finite element model for stud welding was developed in this study using ABAQUS, simulating the temperature field during the welding process and validating the model against experimental temperature measurements. The effects of input current, plate thickness, and multi-stud interactions on steel temperature distribution were systematically investigated. Key findings are summarized as follows.

(1) Infrared thermography validation at welding/ceramic rings showed 5% peak temperature deviation and congruent distribution patterns, confirming model efficacy. This establishes a reliable basis for analyzing residual stress mechanisms.

(2) Consistent thermal evolution patterns emerged radially and through-thickness: rapid heating, followed by cooling decay. Peak temperatures decreased with increasing distance from the welding ring, while time-to-peak exhibited progressive delays due to thermal inertia.

(3) Peak temperatures correlated positively with input current. Temperature gradients were observed to be reduced by lower input current, with a preference being suggested for minimizing thermal stress while ensuring weld quality.

(4) Radial peak temperatures exhibit low sensitivity to plate thickness variations. Temperature gradients along the thickness direction increase significantly with greater plate thickness. Conversely, thinner plates demonstrate more uniform thermal distributions.

(5) Adjacent stud welding induces negligible disturbance to existing stud temperature fields, validating the feasibility of multi-stud welding under standardized spacing protocols.

(6) This study has two main limitations. First, due to the limited availability of stud specifications in practical engineering (only one diameter type was accessible), we were unable to systematically investigate the influence of stud diameter variations on the welding temperature field distribution. Second, the numerical simulation did not fully account for the thermodynamic effects of metallurgical phase transformation latent heat during the welding thermal cycle on temperature field evolution.

## Figures and Tables

**Table 1:** Applicability and limitations of heat source models.

Heat Source Model	Applicability	Limitations
Point Heat Source	Semi-infinite geometries	Significant errors in weld zone calculations
Line Heat Source	Infinite plates	
Surface Heat Source	Infinite rods	
Gaussian Heat Source	Shallow molten pools	Inaccurate for deep molten pools
Double Ellipsoid Source	Deep molten pools	Computationally intensive

**Table 2:** Mesh convergence analysis.

Mesh Size (mm)	Peak Temperature (°C)	Computational Cost
5	1908	Low
4	1950	Moderate
3	1979	Moderate
2	2014	Moderate
1	2019	High
0.5	2021	Very high

**Table 3:** Thermophysical properties of stud and steel.

Temperature	Specific Heat Capacity (10^3^ J/kg·°C)	Thermal Conductivity (10^2^ W/m·°C)	Density (10^4^ kg/m^3^)	Coefficient of Thermal Expansion (10^−5^/°C)
20	0.5	0.5	0.8	1.355
150	0.5	0.5	0.8	1.355
300	0.6	0.4	0.8	1.355
500	0.7	0.3	0.8	1.356
800	0.7	0.3	0.8	1.352
1000	0.7	0.3	0.8	1.36
1200	0.6	0.3	0.8	1.361
1500	0.6	0.3	0.8	1.362
1800	0.7	1.1	0.8	1.364
2000	0.8	1.1	0.8	1.367

**Table 4:** Thermophysical properties of the ceramic ring.

Property	Temperature (°C)	Value
Density (g/cm^3^)	Ambient–High	2.14
Thermal Conductivity (W/m·°C)	25	2.1
	700	3.13
Specific Heat Capacity (J/kg·°C)	Ambient–High	26.1

## Data Availability

The original contributions presented in this study are included in the article. Further inquiries can be directed to the corresponding author.
